# S13-4 Supporting health-enhancing research for cities - peer to peer collaboration, impact and dissemination in the Healthy Cities Helix Community

**DOI:** 10.1093/eurpub/ckad133.066

**Published:** 2023-09-11

**Authors:** Valeria Pulieri, Andreea Petrea

**Affiliations:** Crowdhelix, Ireland; Crowdhelix, Ireland

## Abstract

Support for research and dissemination of project results are often different activities even though they can have a similar target audience. To help support its impact and dissemination activities, while also supporting new collaboration focused on improving the health and wellbeing in cities, the VARCITIES project has launched the Healthy Cities Helix - an international Open Innovation community of specialists in the fields of environment, smart cities, health and related disciplines.

The new Helix is calibrated to support VARCITIES through its various development stages, including post-project. It will accelerate the project’s dissemination, communication and commercialisation strategies, by building a strong, self-sustaining community of like-minded stakeholders that will have access to updates on the project and collaborate with experts in their fields of interest.

The community acts as a virtual accelerator for knowledge transfer and the project’s impact objectives - by linking profiles and outputs from the project to external actors from outside the project consortium who will be part of the Helix. This will not only help enable and facilitate disseminating the results to key STKs, with specific focus on healthy cities, health, and physical activity, but will also drive new forms of international and cross-sectoral partnerships and open innovation activity in these areas.

Currently the community hosts members from 183 organisations across 38 countries, but it can also support researchers to connect in a timely manner to relevant partners by tapping into the 10,000 users profiled on the Crowdhelix platform. This means that users can easily collaborate with people from various fields such as social sciences, citizen science, various areas of health, etc.

The Helix has been set-up as a self-sustaining community hosted on the Crowdhelix Platform beyond the lifetime of the project thereby promoting existing and creating new long-lasting impacts.

